# Shared Features of Endothelial Dysfunction between Sepsis and Its Preceding Risk Factors (Aging and Chronic Disease)

**DOI:** 10.3390/jcm7110400

**Published:** 2018-10-30

**Authors:** Jesus F. Bermejo-Martin, Marta Martín-Fernandez, Cristina López-Mestanza, Patricia Duque, Raquel Almansa

**Affiliations:** 1 Group for Biomedical Research in Sepsis (Bio∙Sepsis), Hospital Clínico Universitario de Valladolid/IECSCYL, Av. Ramón y Cajal, 3, 47003 Valladolid, Spain; xtina.lopez.mestanza@hotmail.com (C.L.-M.); ralmansa@saludcastillayleon.es (R.A.); 2 Centro de Investigación Biomedica En Red-Enfermedades Respiratorias (CibeRes, CB06/06/0028), Instituto de salud Carlos III (ISCIII), Av. de Monforte de Lemos, 5, 28029 Madrid, Spain; 3 Anesthesiology and Reanimation Service, Hospital General Universitario Gregorio Marañón, Calle del Dr. Esquerdo, 46, 28007 Madrid, Spain; patriduque@gmail.com

**Keywords:** aging, chronic disease, endothelium dysfunction, sepsis

## Abstract

Acute vascular endothelial dysfunction is a central event in the pathogenesis of sepsis, increasing vascular permeability, promoting activation of the coagulation cascade, tissue edema and compromising perfusion of vital organs. Aging and chronic diseases (hypertension, dyslipidaemia, diabetes mellitus, chronic kidney disease, cardiovascular disease, cerebrovascular disease, chronic pulmonary disease, liver disease, or cancer) are recognized risk factors for sepsis. In this article we review the features of endothelial dysfunction shared by sepsis, aging and the chronic conditions preceding this disease. Clinical studies and review articles on endothelial dysfunction in sepsis, aging and chronic diseases available in PubMed were considered. The main features of endothelial dysfunction shared by sepsis, aging and chronic diseases were: (1) increased oxidative stress and systemic inflammation, (2) glycocalyx degradation and shedding, (3) disassembly of intercellular junctions, endothelial cell death, blood-tissue barrier disruption, (4) enhanced leukocyte adhesion and extravasation, (5) induction of a pro-coagulant and anti-fibrinolytic state. In addition, chronic diseases impair the mechanisms of endothelial reparation. In conclusion, sepsis, aging and chronic diseases induce similar features of endothelial dysfunction. The potential contribution of pre-existent endothelial dysfunction to sepsis pathogenesis deserves to be further investigated.

## 1. Introduction

Sepsis is a major health problem worldwide. Data exclusively from high-income countries suggests that 50.9 million cases of sepsis occur globally each year, with potentially 5.3 million deaths annually [1]. The global burden of this disease is thought to be much higher, since data on sepsis incidence in low-income and middle-income countries remain scarce. Vascular endothelial dysfunction (ED) is a central event in the pathophysiology of sepsis [2]. ED precedes organ dysfunction and plays an important role in its pathogenesis by increasing vascular permeability, promoting activation of the coagulation cascade, tissue edema and compromising regional perfusion in vital organs [3].

Aging and chronic co-morbidities are recognized risk factors of sepsis. In a report from the American Centers for Disease Control with 246 sepsis patients, the median age was 69 years. Most of the patients in this study (97%) had at least one co-morbidity. A total of 35% had diabetes mellitus, 32% had cardiovascular disease (including coronary artery disease, peripheral vascular disease, or congestive heart failure), 23% had chronic kidney disease, and 20% had chronic obstructive pulmonary disease [4]. Two large epidemiological studies on sepsis, which already use the new SEPSIS-3 criteria to define this disease [5], provide a similar picture of the clinical characteristics of sepsis patients. The studies of Rhee et al. with 173,690 patients [6] and Donnelly et al. with 1080 patients [7] reveal that the mean age of sepsis patients was 66.5 year and 69.7 year respectively. In these large reports, the most frequent co-morbidities present in sepsis patients were those participating of the metabolic syndrome (hypertension, dyslipidaemia, diabetes mellitus), chronic kidney disease, cardiovascular disease, cerebrovascular disease, chronic pulmonary or liver disease, and cancer (Table 1).

It is well-established that aging and the co-morbidities preceding sepsis induce chronic ED. As a result, it is probably that the acute endothelial injury induced by sepsis in aged/chronic disease patients is occurring on an endothelium which is, to a greater or lesser extent, already damaged. Surprisingly, until now there are no studies evaluating the potential influence of the pre-existent ED on the acute ED secondary to sepsis. This review article intends to explore this scenario by identifying common features of ED between sepsis and their preceding risk factors (aging and chronic diseases). We have identified five major features of ED shared between these conditions: (1) increased oxidative stress and systemic inflammation, (2) glycocalyx degradation and shedding, (3) disassembly of intercellular junctions, endothelial cell death, blood-tissue barrier disruption, (4) enhanced leukocyte adhesion and extravasation, (5) induction of a pro-coagulant and anti-fibrinolytic state. Future research works should evaluate if these features could represent a pathogenic trigger of sepsis in aged patients with chronic diseases suffering an infection.

## 2. Search Strategy and Selection Criteria

References for this literature review were identified through searches of PubMed for articles, giving priority to those published in the last 10 years, which constitutes 95% of the articles cited (Table 2). Terms used were “endothelial dysfunction”, “endothelium”, “sepsis”, “aging”, “elderly”. The terms for the chronic diseases associated to sepsis considered in this review are showed in Table 1, and were those reported by Rhee et al. [6] and Donnelly et al. [7]. These are the largest works published to the present date detailing the risk factors associated to sepsis using the new SEPSIS-3 criteria to define this disease [5]. Other terms searched in combination with “endothelial dysfunction” were “repair”, “progenitor cells”, “chemotherapy” and “radiotherapy”.

## 3. The Healthy Endothelium

**Figure 1:**
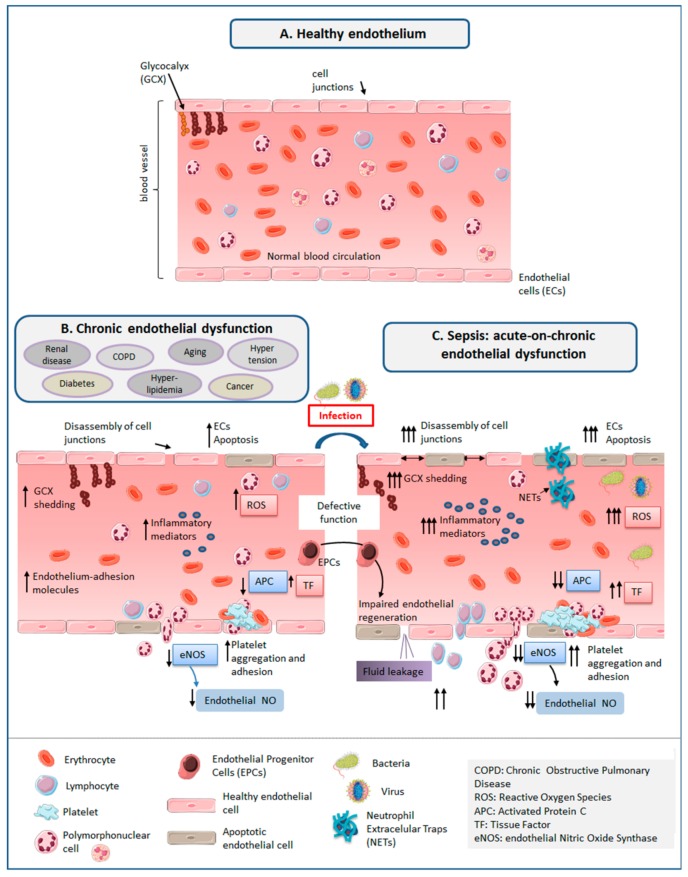
The endothelium in different scenarios: (**A**) Healthy endothelium: the normal vascular endothelium consists of a layer of endothelial cells with the glycocalyx on the luminal side. It prevents microorganisms to enter into tissues, exerting in addition a natural anticoagulant action that prevents from uncontrolled activation of the coagulation system; (**B**) Endothelial dysfunction (ED) induced by aging and chronic disease: senescence and the comorbidities preceding sepsis are associated to the presence of a chronic status of increased oxidative stress and inflammation, which induces glicocalyx degradation, apoptosis of endothelial cells, disassembly of endothelial cell junctions, and increased expression of molecules which promotes leukocyte adhesion to endothelial cells. In turn, these diseases induce a pro-coagulant and anti-fibrinolytic state with diminished activation of protein C and increased production of tissue factor. Decrease in the production of nitric oxyde by the endothelial nitric oxide synthase promotes platelet aggregation and adhesion. Finally, these diseases impair production and function of Endothelial Progenitor Cells, impairing endothelial regeneration; (**C**) ED in sepsis: sepsis induces similar features of ED to those caused by aging and chronic diseases, inducing oxidative stress and inflammation, release of NETs and proteases by neutrophils, leading to fluid and cell leakage, hypotension, microvascular thrombosis, inadequate organ perfusion, organ failure and shock in the most severe cases. In addition, bacterial toxins can breach the endothelial barrier, by directly killing endothelial cells (ECs), weakening the cytoskeleton within ECs, and breaking the junctions between ECs. Acute challenges such as the aggression induced by surgery, trauma or hypervolemia could contribute to facilitate or enhance ED in patients facing an infection. Images for representing cells were taken from “Smart Servier Medical Art” (https://smart.servier.com/). NO: nitric oxide.

The vascular endothelium constitutes a semi-permeable barrier lining the inner surface of blood vessels (Figure 1). It controls the exchange of fluids, solutes, plasma proteins and leucocytes, by opening and closing the cell junctions composing it in a coordinated manner [69]. The normal vascular endothelium consists of a layer of endothelial cells (ECs), supported on a basement membrane, with the glycocalyx on the luminal side [45]. It prevents microorganisms to enter into tissues, exerting in addition a natural anticoagulant action that prevents from uncontrolled activation of the coagulation system.

### 3.1. Glycocalyx (GCX)

It is an organized layer of sulfated proteoglycans, hyaluronan, glycoproteins, and plasma proteins that adhere to a surface matrix which coats the luminal surface of the endothelium. It serves as a protective barrier between the flowing blood and the vessel wall, contributing to maintain the endothelial barrier to fluid and protein, to regulate leukocyte-endothelial adhesion and to inhibit intravascular thrombosis [17].

### 3.2. Endothelial Cells (ECs) 

ECs line our vasculature, as a one continuous layer resting on a basement membrane formed by collagen, laminins, nidogens/entactins, and the proteoglycan perlecan. Endothelial cells lining the vessel wall are connected by adherens junctions (mainly composed of vascular endothelial (VE)-cadherin), tight junctions (predominantly consisting of occludins and claudins) and gap junctions [2,70], which prevent leukocyte emigration and vascular leak [2]. Embedded in the basement membrane and outside it is a non-continuous layer of cells known as pericytes, which are thought to play a role in angiogenesis [45].

## 4. ED in Sepsis

Sepsis causes acute ED, inducing a pro-adhesive, pro-coagulant and anti-fibrinolytic state in ECs, altering hemostasis, leukocyte trafficking, inflammation, barrier function, and microcirculation [30] (Figure 1).

### 4.1. Increased Oxidative Stress and Systemic Inflammation

There are a number of mediators participating in the “molecular storm” occurring in sepsis that initiate and amplify injury to the endothelium. Between these molecules are bacterial endotoxins/pathogen-associated molecular patterns (PAMPs), cytokines, bradykinin, histamine, platelet-activating factor (PAF), vascular endothelial growth factor (VEGF), fibrin degradation products and reactive oxygen species (ROS) such as hydrogen peroxide, hydroxyl anions, and superoxide [3,8,9,10]. In turn, the endothelium it is not just a passive element suffering the aggression during sepsis, but also produces chemokines to attract immune cells, boosting the inflammatory response [10].

### 4.2. GCX Degradation and Shedding

The “cocktail” of pro-inflammatory and pro-oxydative molecules induced by sepsis promotes shedding of the GCX [2,3,18]. Release of damage-associated molecular patterns (DAMPs) such as degradation products of the endothelial GCX (i.e., heparan sulfates) or components of Neutrophil extracellular traps (NETs) amplifies this deleterious response [17,18].

### 4.3. Disassembly of Intercellular Junctions, Endothelial Cell Death, Blood–Tissue Barrier Disruption

The marked pro-inflammatory and oxidative response occurring in sepsis induces also the formation of gaps between ECs by disassembly of intercellular junctions [2,3,18]. NETs released from dying neutrophils induce death of ECs, an effect mediated by NETs-related proteases and cationic proteins such as defensins and histones [30,31]. Bacterial toxins can breach the endothelial barrier, by directly killing ECs, weakening the cytoskeleton within ECs, and breaking the junctions between ECs [9].

### 4.4. Enhanced Leukocyte Adhesion and Extravasation

Glycocalyx shedding exposes the endothelium to leukocyte adhesion [18]. Pro-inflammatory cytokines induce expression of molecules such as *p*-selectin, E-selectin, intercellular adhesion molecule 1 (ICAM-1) or vascular cell adhesion molecule 1(VCAM-1) that allow adhesion of activated immune cells to the vascular wall and promote transendothelial migration into surrounding tissues [3]. Activated neutrophils from sepsis patients adhered to endothelium mediate profound loss of endothelial barrier integrity [46]. The proteases released by activated neutrophils could contribute to degrade junctional proteins [45]. Extravased neutrophils induce tissue damage producing potentially destructive enzymes and oxygen-free radicals.

### 4.5. Induction of A Pro-Coagulant and Anti-Fibrinolytic State

In sepsis there is also a significant increase in the production of nitric oxide (NO, a potent vasodilator) mediated by the inducible nitric oxide synthase (iNOS) [3,54]. In contrast, there is an important decrease in the production of NO by endothelial nitric oxide synthase (eNOS), which impairs direct vasodilatation, and promotes platelet and leukocyte adhesion [8]. Down-regulation of endothelial expression of thrombomodulin and endothelial protein C receptors translates into diminished activation of the activated protein C [30]. ECs release the procoagulant glycoprotein TF (tissue factor), whereas their synthesis of TF pathway inhibitor is inhibited [30]. The activation of platelets and the coagulation cascade causes microvascular thrombosis [17]. In addition, NETs provide a scaffold for thrombus formation, promoting hypercoagulability in patients with sepsis [30]. The association of TF with NETs could target thrombin generation and fibrin clot formation at sites of infection/neutrophil activation, with active thrombin leading to increased platelet activation [55]. Acute vascular dysfunction and leakage contribute to hypotension, inadequate organ perfusion, local hypoxia, ischemia and ultimately, to organ failure, acute respiratory distress syndrome, shock and death in the most severe patients [8,56].

## 5. ED Associated to Aging and Chronic Disease

The same features of ED induced by sepsis are also induced by aging and chronic disease (Figure 1).

### 5.1. Increased Oxidative Stress and Systemic Inflammation

Aging is characterized by the presence of increased endothelial oxidative stress, as a result of augmented production from the intracellular enzymes NADPH oxidase and uncoupled eNOS, as well as from mitochondrial respiration in the absence of appropriate increases in antioxidant defenses [11]. Nitroso-oxydative stress contributes to ED associated with diabetes [12]. Vascular oxidative stress and inflammation are major determinants of ED in atherosclerosis and cardiovascular diseases [13]. Inflammation and free radical formation contributes also to the pathogenesis of hypertension and cancer [14]. In patients with Chronic Obstructive Pulmonary Disease (COPD), chronic inflammation not only impacts on lung parenchyma, but potentially also involves the endothelium of blood vessels, which makes it a systemic disease [15]. Inflammation and oxidative stress play major roles in the pathogenesis of ED in liver cirrhosis [16].

### 5.2. GCX Degradation and Shedding

Untreated hypertension, diabetes mellitus and hypercholesterolemia are associated to a reduced endothelial GCX thickness [19,20,21]. Hyperglycaemia and oxidised low-density lipoprotein (LDL) causes GCX dysfunction [22]. GCX degradation is an initial event in atherosclerosis, promoting lipid deposition in the vessel wall [23]. Patients with chronic renal disease under dialysis have an impaired GCX barrier and shed its constituents into blood [24]. Cigarette smoking (the main cause of COPD) compromises endothelial GCX integrity [25]. Patients with end-stage liver disease show marked increased concentration of syndecan-1 in plasma, a marker of GCX shedding [26]. Elevated haematocrit, a risk factor for stroke and myocardial infarction, could induce a reduction in GCX thickness [27]. Patients with heart failure with reduced ejection fraction have increased levels of the GCX shedding marker median hyaluronic acid [28]. Lacunar stroke patients with white matter lesions show compromised GCX barrier properties [29].

### 5.3. Disassembly of Intercellular Junctions, Endothelial Cell Death, Blood-Tissue Barrier Disruption

Sedentary aging enhances endothelial cell senescence. Senescent ECs show a pro-oxidant phenotype with increased ROS production, which promote endothelial injury [11]. Tight junction structure and barrier integrity is significantly impaired in senescent ECs [32]. LDL from patients with hypercholesterolaemia are inflammatory to microvascular endothelial cells, impairing in addition endothelial tight junction expression [33]. Activation of endothelial inflammasomes due to increased free fatty acids produces high mobility group box protein-1, HMGB1, which disrupts inter-endothelial junctions and increases paracellular permeability of endothelium [34]. High glucose concentrations induce disruption of endothelial adherens junctions mediated by protein kinase C-β-dependent vascular endothelial cadherin tyrosine phosphorylation [35]. Patients with chronic kidney diseases have increased levels of anti-endothelial cell antibodies, and decreased expression of both adherens and tight junction proteins VE-cadherin, claudin-1, and zonula occludens-1 [36]. Cigarette smoking disrupts intercellular adhesion molecules between ECs and induces their apoptosis [37]. In COPD patients, circulating anti-endothelial cell antibodies along with chronic oxidative and inflammatory stress induces apoptosis of ECs [38]. Cancer induces also disruption of endothelial junctions, in particular adherens junctions [39]. Anticancer chemotherapy may induce systemic damage of vascular endothelium related to massive cell loss and alterations of endothelial function [40]. Radiotherapy causes premature senescence, apoptosis of endothelial cells and increased vascular permeability [41]. Endothelial barrier is also altered in cardiovascular disease. Chemical modification of tubulin caused by cardiometabolic risk factors and oxidative stress leads to reorganization of endothelial microtubules, destabilizing vascular integrity and increasing permeability, which finally results in increasing cardiovascular and cerebrovascular risk [42]. Intravascular albumin is important for maintaining vascular integrity, since it contributes to preserve normal capillary permeability [43] and the GCX structure [44]. Patients with malnutrition, liver disease or nephrotic syndrom could present hypo-albuminemia.

### 5.4. Enhanced Leukocyte Adhesion and Extravasation

Aging is associated to stiffening of extracellular matrix within the intima, which promotes EC permeability and leukocyte extravasation [47]. Hypertension induces vascular wall injury and remodeling, a process which involves recruitment of leukocytes to the endothelium [14]. Glycocalix degradation in atherosclerosis facilitates the interaction between ECs and leukocytes [23]. Hyperglycemia induces activation of NF-κB in ECs, leading to an increased production of adhesion molecules, leukocyte-attracting chemokines and cytokines activating inflammatory cells in the vascular wall [48]. In chronic kidney disease, leukocytes acquire an adhesive phenotype, by mechanisms mediated by hypoxia and by cytokines released from ischemic renal endothelium [49]. In patients with COPD, fibrinogen is increased and stimulates the adhesion of platelets and white blood cells to the vessel wall [15]. Tobacco nicotine causes a loss of functional integrity of endothelium by causing vasospasm, stimulating the adhesion of leukocytes [50]. Up-regulation of selectins seems to be a central event in metastatic progression in cancer, proteins which mediate tethering and rolling of leukocytes to the vascular endothelium. Regarding cancer treatment, radiotherapy leads to increased endothelial cell activation andexpression of VCAM-1, ICAM-1, PECAM-1, E-selectin and *p*-selectin, which promotes adhesion of leukocytes [41]. Chronic liver disease is characterized by upregulation of endothelium-adhesion molecules such as CD11b in circulating leukocytes in blood [51]. Leukocyte activation, adhesion and accumulation in the endothelium are events playing an important role in the pathogenesis of different cardiac diseases (myocarditis, cardiomyopathy, cardiac hypertrophy and failure, and ischemic heart disease) [52] and in ischemic cerebrovascular disease [53].

### 5.5. Induction of A Pro-Coagulant and Anti-Fibrinolytic State

Senescent ECs show reduced eNOS activity, which impairs their ability to inhibit platelet aggregation [57]. Hypertension is associated to ED leading to attenuated NO formation because of direct oxidative modification of eNOS [58]. GCX degradation in atherosclerosis causes ECs to reduce their expression of eNOS [23]. Oxidative stress associated with hyperglicemia induces eNOS uncoupling and reduce NO production [12]. Hyperglycemia, excess free fatty acid release and insulin resistance in diabetes mellitus induces platelet hyperactivity [48]. Additionally, hyperglycemia generates a prothrombotic state by the increased production of lesion-based coagulants, such as tissue factor, and the inhibitors of fibrinolysis, such as PAI-1 [48]. Patients with chronic renal disease can show either coagulation defects and endothelial cell damage leading to a thrombophilic state, which is characterized by the presence of high plasma concentration of fibrinogen, D-dimer, thrombin–antithrombin complex, coagulation factor VII, vWF, thrombomodulin and PAI-1 [59]. Patients with COPD or liver cirrhosis have impaired eNOS activity also [15,16]. Smoking along with the maintained pro-inflammatory state in COPD induce a thrombotic effect, by increasing platelet activation and triggering coagulation cascade [15]. Cancer may result in activation of coagulation and endothelial cell perturbation, leading to coagulopathies, a prothrombotic state and microvascular dysfunction, by mechanisms involving tissue factor-mediated thrombin generation, down regulation of endothelial cell-associated physiological anticoagulant pathways, deranged fibrinolysis and dysfunctional ECs [60]. This pro-coagulating phenotype in cancer could also be favored in chemo and radiotherapy [41,60]. Patients with chronic liver disease show hyperhomocysteinaemia, a disorder of methionine metabolism, which has been suggested to cause endothelial injury and atherothrombotic vascular disease by several mechanisms involving oxidative stress, altered production of NO and impaired platelet-modulating activity [61]. Coronary heart disease associated with hypertension is characterized by reduced endothelial NO synthesis [62]. Endothelial dysfunction and coagulation are also involved in the pathogenesis of ischaemic stroke [63].

## 6. Impact of Aging and Chronic Disease on the Mechanisms of Endothelial Repair

Endothelial cell injury is mitigated by the reparative activity of bone marrow-derived endothelial progenitor cells (EPCs). As recently described, EPCs could have a role in determining the prognosis of patients with sepsis [64]. Cell senescence impairs the regenerative capacity of ECs. Age impairs migration of endothelial progenitor cells (EPCs) reducing their ability to contribute to vascular repair [65]. Chronic diseases preceding sepsis decrease EPC availability and/or mobilization. The absolute number, or functional capacity of EPCs, has been shown to be reduced in several disease states including diabetes, hypertension, hypercholesterolemia, and chronic kidney disease [66]. Circulating EPCs from COPD are dysfunctional, displaying impaired angiogenic ability and increased apoptosis [67]. Radiotherapy lowers the number of circulating EPCs in cancer survivors [68]. The consequences of chemotherapy on EPCs at the long term are unknown.

## 7. Link between Chronic Endothelial Dysfunction and Sepsis: “Proof of Concept” Studies

The evidence available from the literature shows that sepsis and their preceding risk factors (aging and chronic diseases) share common features of ED, characterized by loss of the endothelium`s tightly regulated balance and the presence of sustained EC activation. As occurs in sepsis, aging and chronic ED promotes a pro-inflammatory, pro-oxidative and pro-coagulation status in the blood vessels, favoring vasomotor tone alterations, platelet activation and leukocyte adhesion and transmigration. In addition, aging and chronic diseases impair the regenerative ability of the endothelium. Aging and chronic ED thus contribute to generating a basal degree of organ failure [14]. All of this draws a scenario of “endothelial frailty” preceding sepsis. The presence of previous ED could represent thus a predisposing factor for developing new ED when the patient suffers an infection (a phenomenon of “Acute-on-Chronic ED”), contributing to generate new organ failure in these patients, or which is the same, sepsis (Figure 1).

**Figure 2:**
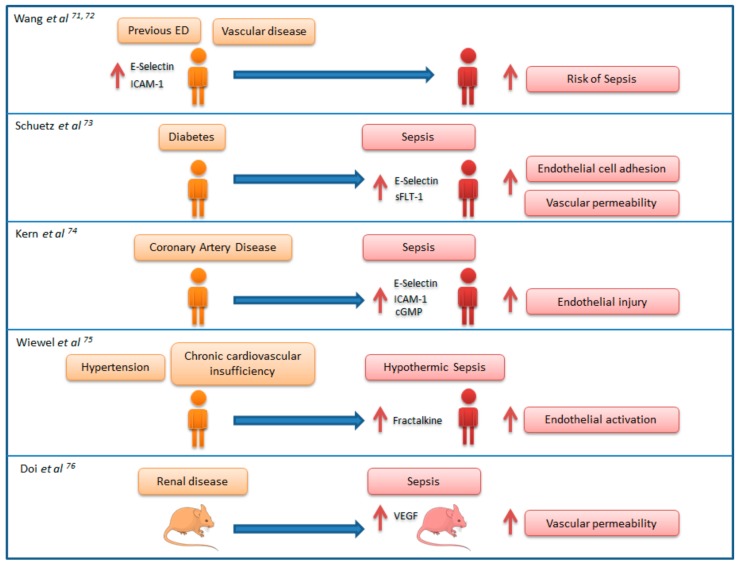
Proof of concept studies supporting the connection between chronic ED and sepsis. Images for representing mice were taken from “Smart Servier Medical Art” (https://smart.servier.com/). ICAM-1: intercellular adhesion molecule 1; VEGF: vascular endothelial growth factor; cGMP: cyclic guanosine monophosphate; sFLT-1: fms-like tyrosine kinase-1.

There is a number of “proof of concept” studies supporting the link between chronic ED and sepsis (Figure 2). In an elegant study on a cohort of individuals with no sepsis living in the community, Wang et al. showed that high basal levels of endothelial activation makers in serum (E-selectin and ICAM-1) conferred a higher risk of developing future episodes of sepsis. As the authors discuss in this article, basal elevation of these biomarkers may indicate individuals that are prone to develop a higher degree of ED in the face of acute infection [71]. In a large epidemiological study, the same authors identified an association between the antecedent of vascular disease and risk of developing sepsis, proposing endothelium dysfunction as the physiopathological link between these two conditions [72]. Schuetz P. et al. showed in a cohort of sepsis patients, that compared to non-diabetic patients, those patients with diabetes had higher levels of circulating E-selectin and fms-like tyrosine kinase-1 (a VEGF signaling protein contributing to vascular leak), and propose developing future studies to investigate whether patients with diabetes suffering from sepsis show an enhanced activation of the endothelium [73]. Similarly, Kern et al. evidenced that sepsis patients with preexisting coronary artery disease shows increased endothelial injury (indicated by the elevated levels of ICAM-1, E-selectin, and cGMP) compared to those with no antecedent of this disease [74]. Wiewel et al. showed that the antecedent of hypertension and chronic cardiovascular insufficiency is associated with an increased risk of hypothermic sepsis, an especially severe form of this disease, and propose a fractalkine-related endothelial activation mechanism to explain the link between these cardiovascular risk factors and sepsis [75]. Using an animal model, Doi et al. demonstrated that septic mice with pre-existing renal disease had significantly higher mortality, vascular permeability, plasma vascular endothelial growth factor levels (VEGF; a growth factor known to enhance vascular permeability), and more severe septic shock when compared to septic mice without pre-existing disease [76].

## 8. Other Factors Inducing ED before Sepsis

In addition to aging and chronic disease, there are other factors such as critical illness, surgery, trauma or hypervolemia which induce ED preceding sepsis [77,78,79]. Increasing evidence supports that this previous ED could facilitate the incidence of sepsis. Vassiliou A.G. et al. in a cohort of initially non-septic critically-ill patients, found that the presence of elevated biomarkers of ED at ICU admission (Soluble E- and *p*-selectin levels) predicted sepsis development [80]. In the same scenario (non-septic critically-ill patients), Vassiliou A.G. et al. have also demonstrated that soluble endothelial protein C receptor levels at intensive care unit (ICU) admission (a protein regulates the protein C anticoagulant activity) are elevated in the subjects who will subsequently become septic [81]. In a recently published article, Wei S. et al. demonstrate that a high degree of ED following traumatism (as assessed by levels of Syndecan 1 in serum) is associated with an increased risk of developing sepsis [79]. An old work from Ikegami K. et al. showed that the degree of endothelial cell injury in patients suffering blunt trauma (as quantified by the soluble thrombomodulin levels) predicted the incidence of sepsis [82].

## 9. Implications for Clinical Practice and Future Research

### 9.1. Quantifying ED to Predict or Early Detect Sepsis in Infected Patients

When an aged individual with chronic disease shows signs of infection, monitoring ED could help to predict or early detect sepsis [17]. ED could be profiled “in vivo” using non-invasive image techniques. Examples are flow-mediated vasodilation [66], intravital microscopy or hand-held video images of sublingual microcirculation [3]. The other approach to evaluate ED is using biomarkers. Biomarkers of ED have been reviewed elsewhere [83,84,85,86] and include markers of endothelial glycocalyx degradation such as heparan sulfate, chondroitin sulfate, hyaluronic acid and syndecan [87], markers of endothelial cell activation such as endocan [88] or Angiopoietin-2 [85], cell adhesion molecules such as selectins [85], ICAM-1 and VCAM-1 [88], vasoactive peptides such as midregional proadrenomedullin [89] and midregional proatrial natriuretic peptide [84], coagulation inhibitors such as thrombomodulin [84], molecules with vasoconstrictor and vasopressor activity such as endothelin [85], growth factors such as vascular endothelial growth factor (VEGF) [85], and circulating endothelial cells [66] as some of the most relevant. As previously proposed, a multimarker strategy could represent the best approach to cover the different features of endothelial injury [84]. Methods such as the ultra-high performance liquid chromatography coupled to mass spectrometry-based multiple reaction allows to simultaneously profile multiple endothelial biomarkers [84]. Development of “point of care” approaches for profiling ED at the community, the emergency room or at the hospital ward could contribute to diagnose sepsis earlier [90].

### 9.2. Endothelium-Protective Therapies for the Prevention and Treatment of Sepsis

In elderly patients or those with chronic diseases who suffer an infection, combining antimicrobials with drugs protecting the endothelium could prevent the development of sepsis or improve outcome once sepsis is present. Many potential treatment options to prevent or treat ED have been proposed, [13,44,86,91], but probably the most successful example to the present date is the trial performed by Marik et al. These authors have demonstrated that the early administration of intravenous vitamin C, corticosteroids and thiamine prevent progressive organ dysfunction and reduce mortality of patients with sepsis [92]. In an elegant “in vitro” work, the group of Dr. Marik demonstrated that Hydrocortisone and Ascorbic Acid exert a synergistic effect preventing and repairing endothelial barrier dysfunction induced by Lipopolysaccharide [93]. Works on cellular and animal models will help to elucidate the potential role of endothelium-protective drugs for the prevention and treatment of sepsis in the infected host.

### 9.3. Other Potential Research Avenues

**Figure 3:**
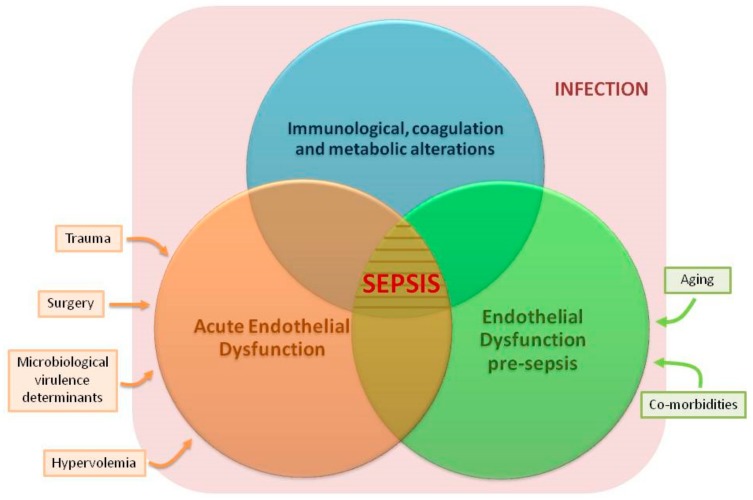
Potentially synergistic factors contributing to sepsis. Blue circle: immunological, coagulation or metabolic alterations previous to sepsis or induced by sepsis. Green circle: chronic endothelial dysfunction (ED) pre-sepsis caused by aging and chronic diseases. Orange circle: acute ED induced by sepsis secondary to microbial aggression, to the dysfunctional host response to infection, hypervolemia, trauma, surgery, and/or to other acute insults facilitating sepsis.

(1) Developing future studies evaluating the specific impact of aging and each one of the chronic diseases discussed here on the endothelium will help to better understand their individual contribution to sepsis. Animal and cellular models could be very useful to this regard. (2) Our review focused on those co-morbidities linked to sepsis in high income countries. In low-middle income countries, the epidemiological profile of sepsis is different. Sepsis patients are younger there, but they suffer frequently from a number of conditions also linked to ED, such as malnutrition, malaria and HIV infection. Evaluating the chronic impact of these conditions on the endothelium and also on the risk of developing sepsis deserves further research efforts. (3) Our review addresses only vascular ED. There is a total lack of information in the literature on the impact of sepsis, aging and chronic disease on the lymphatic vessels’ endothelium. The lymphatic system could have important implications in sepsis physiopathology, since it is the main avenue for circulation of dendritic cells and lymphocytes. (4) Finally, studies evaluating the interactions between chronic endothelial, immunological, coagulation and metabolic dysfunction will help to better understand the pathogenesis of sepsis (Figure 3).

## 10. Conclusions

Sepsis, aging and chronic disease induce similar features of ED. There is growing evidence supporting that pre-existing ED could represent a predisposing factor for sepsis, facilitating the induction of acute ED in those patients facing an infection. Further studies should elucidate whether monitoring ED in elderly individuals with chronic disease could help to predict or to early identify sepsis, and also if administration of drugs improving ED could have a beneficial effect to prevent or treat this disease.

## Figures and Tables

**Table 1:** Mean age, sex and major co-morbidities associated to sepsis.

Rhee et al. (*n* = 173,690)		Donnelly et al. (*n* = 1080)	
Age (mean in years)	66.5	Age (mean)	69.7
Sex (male)	57.6%	Sex (male)	59.2%
Diabetes	35.7%	Hypertension	74.5%
Chronic pulmonary disease	30.9%	Dyslipidaemia	67.3%
Renal disease	26.8%	Diabetes	41.8%
Congestive heart failure	25.4%	Chronic kidney disease	31.5%
Cancer	19.7%	Myocardial infarction	24.4%
Dementia or cerebrovascular disease	10.3%	Chronic lung disease	17.4%
Liver disease	10%	Stroke	12.6%

These data correspond to the studies of Rhee et al. [6] and Donnelly et al. [7]. Co-morbidities are showed by their observed prevalence in each study.

**Table 2:** References describing the features of endothelial dysfunction (ED).

Features of ED	Sepsis	Aging/Chronic Disease
Increased oxidative stress and systemic inflammation	[3,8,9,10]	[11,12,13,14,15,16]
Glycocalyx degradation and shedding	[2,3,17,18]	[19,20,21,22,23,24,25,26,27,28,29]
Disassembly of intercellular junctions, endothelial cell death, blood-tissue barrier disruption	[2,3,9,18,30,31]	[11,32,33,34,35,36,37,38,39,40,41,42,43,44]
Enhanced leukocyte adhesion and extravasation	[3,18,45,46]	[14,15,23,41,47,48,49,50,51,52,53]
Induction of a pro-coagulant and anti-fibrinolytic state	[3,8,17,30,54,55,56]	[12,15,16,23,41,48,57,58,59,60,61,62,63]
Impairment in the mechanisms of endothelial repair	[64]	[65,66,67,68]
